# Misdiagnosed murine typhus in a patient with multiple cerebral infarctions and hemorrhage: a case report

**DOI:** 10.1186/s12883-015-0383-4

**Published:** 2015-07-30

**Authors:** Ziqi Xu, Xiongchao Zhu, Qunying Lu, Xia Li, Yewen Hu

**Affiliations:** Department of Neurology, The First Affiliated Hospital of the College of Medicine, Zhejiang University, No. 79 Qingchun Road, Hangzhou, 310003 China; Department for Microbiology, Zhejiang Province Center for Disease Control and Prevention, Hangzhou, China

**Keywords:** Endemic or murine typhus, Cerebral infarction, Cerebral hemorrhage, Multiple lesions

## Abstract

**Background:**

Rickettsias cause a wide spectrum of tick-, flea-, or mite-borne infections. Rickettsial infections have no classical manifestations and can often lead to encephalitis, which can be fatal if improperly diagnosed.

**Case presentation:**

A 74-year-old male farmer was admitted to the hospital with fevers and a headache that had lasted for 10 days, followed by 4 days of unconsciousness, and his condition continued to deteriorate. Images showed multiple acute lesions in the brain stem, and bilateral cerebral and cerebellar hemispheres. He was finally diagnosed with endemic typhus and treated with antibiotics that resulted in improvement.

**Conclusion:**

The present report describes a patient with a rickettsial infection and subsequent deterioration to coma because of an initial misdiagnosis. Because of the similarity to other infectious diseases, physicians should be more vigilant towards the history and radiologic results to ensure early detection and avoid complications which may prove to be fatal.

## Background

Rickettsial infections are caused by a wide spectrum of tick-, flea-, or mite-borne infections, the distribution of which is directly dependent on the arthropod vector or vectors [1]. The most common symptoms of rickettsial infections include fevers, rashes, headaches, dizziness, myalgias, arthralgias, and anorexia, with the classic clinical triad being fevers, rash, and unremitting headache [2]. Currently, there are no detailed data on the epidemiology of typhus, which is sometimes a localized outbreak [3, 4]. Previous Chinese epidemiologic data have shown that the prevalence of typhus fluctuates between 0.25 and 0.49 per 10,000 population [5]. Endemic typhus caused by *Rickettsia typhi* is the most common type of rickettsial infection in China and is considered to be self-limiting [6, 7]. Herein we describe the misdiagnosis of a severe case of endemic typhus that presented with multiple, diffuse lesions in the cerebral white matter.

## Case presentation

**Fig. 1:**
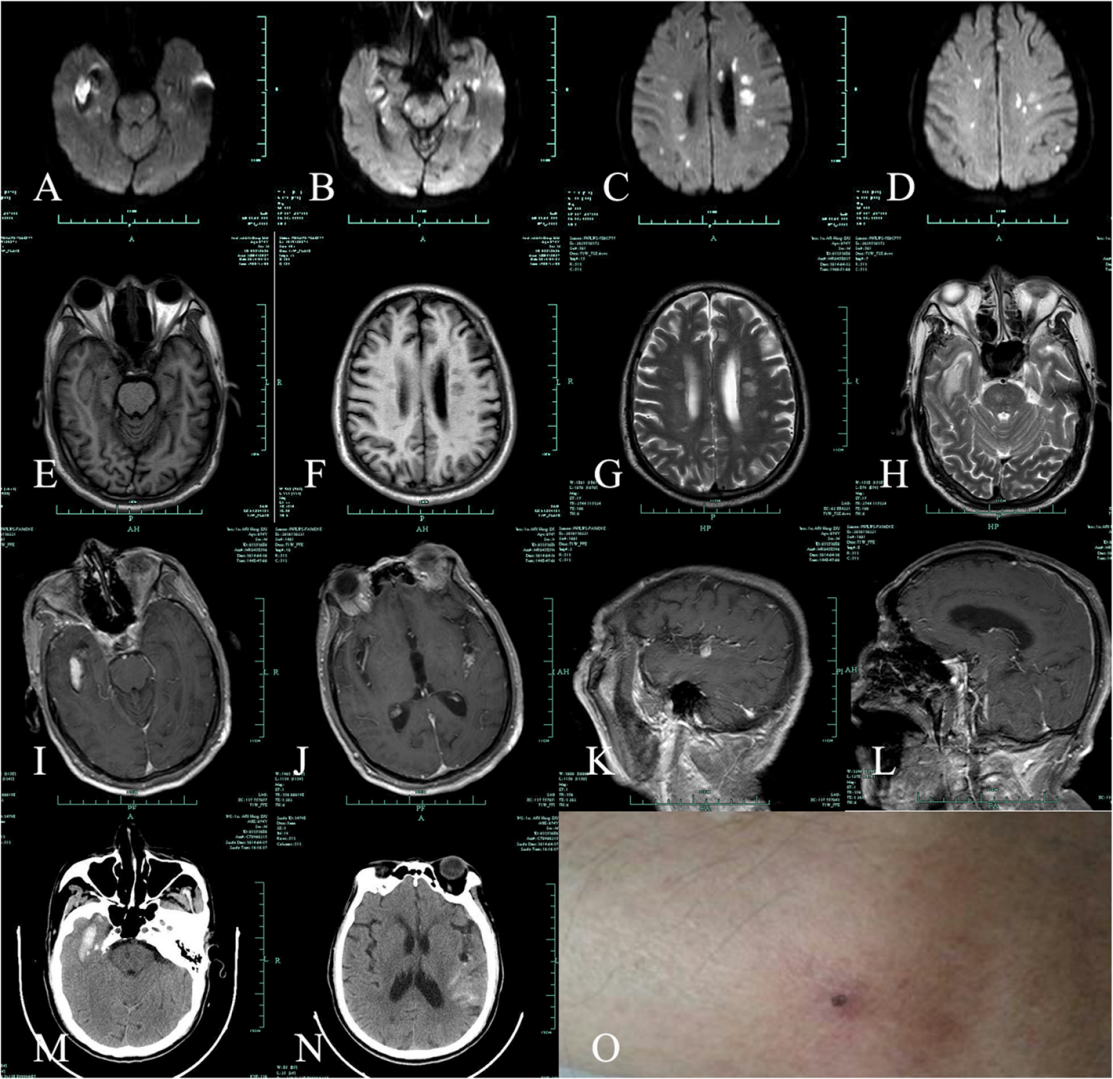
An initial cranial MR image showing hemorrhage in the right temporal lobe, left temporal parietal subarachnoid, and multiple infarcts throughout the deep white matter. **a**-**d**: A diffusion-weighted image (DWI) shows increased intensity in central of the lesion with reduced intensity surrounding the lesion in the right temporal lobe. Multiple hyperintense lesions can be seen in the brain stem, bilateral cerebral, and cerebellar hemispheres. **e**-**f**: T1-weighted image (T1WI) shows isointensity lesions in the right temporal lobe and multiple hypointense lesions throughout deep white matter. **g**-**h**: T2-weighted image (T2WI) shows hyperintense areas in the right temporal and multiple hyperintense lesions throughout the deep white matter. **i**-**l**: enhanced T1-weighted image shows no enhanced lesions or dural meningeal enhancement. **m**-**n**: cranial CT shows right temporal lobe hemorrhage and left temporal parietal subarachnoid hemorrhage. **o**: an eschar present on the left calf

**Fig. 2:**
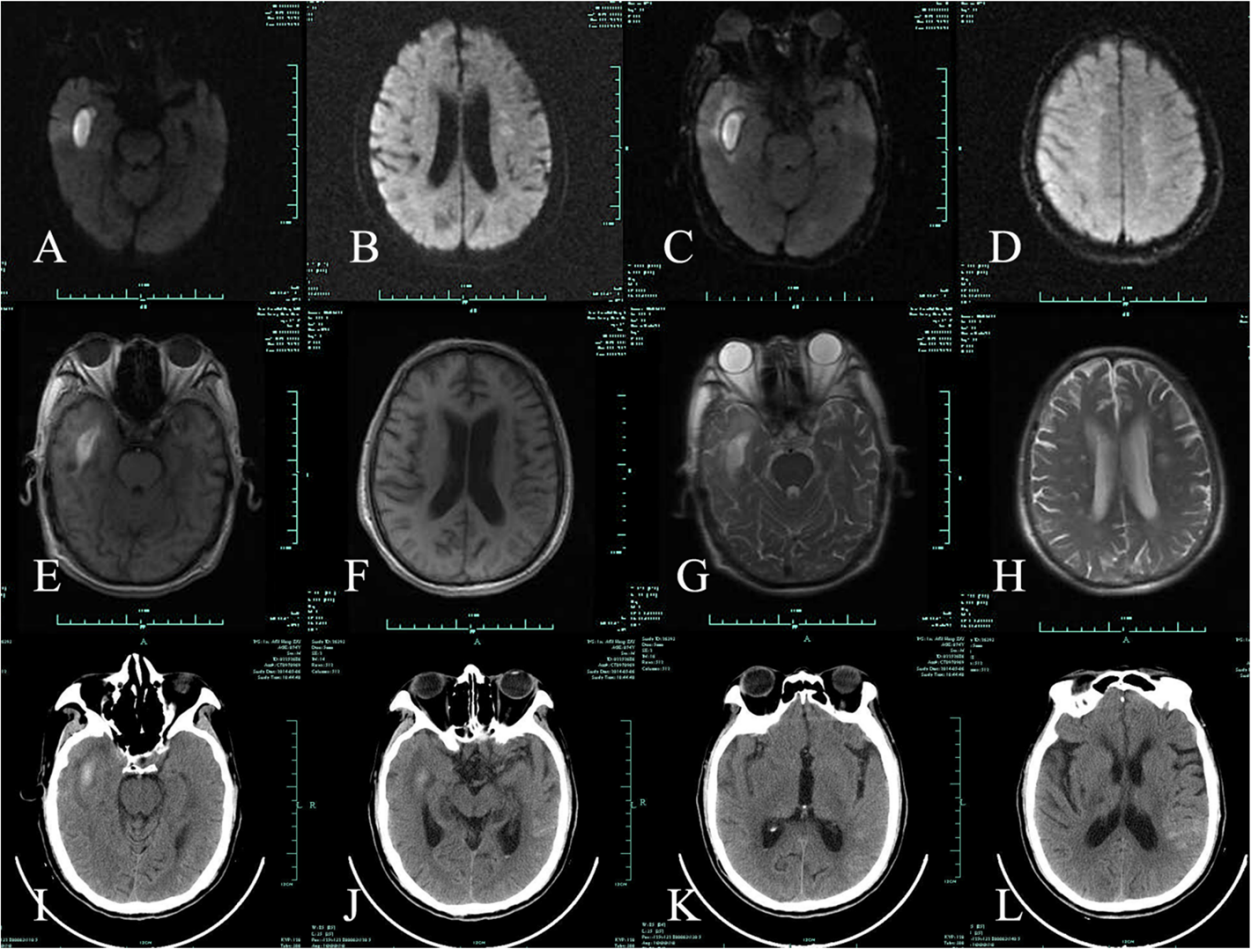
A repeat MR image following treatment showing a reduced number of intracranial lesions and subsequent absorption of cerebral hemorrhage. **a**-**b**: DWI shows hyperintense lesions in the right temporal lobe and a reduced number of hypointense lesions. **c**-**d**: fluid-attenuated inversion recovery (FLAIR) shows hyperintense lesions in the right temporal lobe and hyperintense lesions in the deep white matter. **e**-**f** (T1WI) and **g**-**h**(T2WI): also shows hyperintensity lesions in the right temporal lobe reduced numbers of leseions in the deep white matter. **i**-**l**: cranial CT shows high-density lesions in the right temporal lobe and left temporal parietal subarachnoid; the volume of cerebral hemorrhage has been reduced

A 74-year-old male farmer from Tonglu County in Zhejiang province was admitted to the hospital for acute onset of impaired consciousness preceded by fevers and headaches lasting for days. The patient was healthy until 10 April 2014 when he presented with severe headaches, fevers, and dizziness associated with nausea. The patient had a 10-year history of hypertension and a 40-year history of alcohol and tobacco use. A physical examination and chest X-ray performed at a local clinic were unremarkable. The patient was treated symptomatically for 10 days, but without improvement. The emergency room evaluation revealed poor orientation to time, place, and person. He was febrile with a temperature of 38–39 °C. A cranial MRI showed multiple acute lesions with hyperintensity on diffusion weight imaging (DWI) involving the brain stem, and bilateral cerebral and cerebellar hemispheres (see Fig. 1a-h). Encephalitis with unknown pathogens was suspected. Empiric therapy with intravenous cefoperazone sulbactam and ribavirin was initiated. On 24 April the patient was admitted to the Neurology ward for further evaluation. A more comprehensive diagnostic work-up was carried out. A color Doppler cardiac examination was considered normal, thus ruling out subacute infective endocarditis. A lumbar puncture was performed on the hospital day 2; the CSF findings were non-diagnostic and a serum sample was obtained; the results are shown in Table 1. Despite aggressive antibiotic treatment, the patient’s neurologic condition worsened. A neurologic examination was notable for mild coma with neck stiffness, increased muscle tone throughout the body, and an extensor plantar response bilaterally. A repeat MRI with enhancement showed an increased number of intracranial lesions (Fig. 1i-l). A cranial CT done the same day indicated a right temporal lobe and left temporal parietal subarachnoid hemorrhage (Fig. 1m-n). On hospital day 3, the patient’s daughter inadvertently mentioned that her father had been to the family cemetery, which was located in a wild, undeveloped area, 10 days prior to the onset of his disease. He subsequently complained of an itch resulting from a bug bite. An eschar was noted involving the left leg (Fig. 1o). On the basis of a detailed medical history and characteristic skin changes, a rickettsial infection was tentatively diagnosed and minocycline therapy was initiated. Two weeks after the onset of symptoms, 5 mL of blood was collected from the patient with the consent of his relative and transported to the Department of Rickettsiology of the Institute of Communicable Disease Control and Prevention in Zhejiang province for testing. Serum IgG to *R. typhi*, *R. conorii*, *O. tsutsugamushi*, and *A. phagocytophilum* were detected using the IgG IFA Kit (Fuller Laboratories and Focus Diagnostics, Cypress, CA, USA). The baseline titer for rickettsial antibodies (IgG) was 1:128. Two weeks later, the rickettsial IgG antibody titer was 1:2048, and a negative serum parasite antibody test was obtained (see Table 1). DNA was extracted from blood samples using a Universal DNA Extraction Kit (Takara Biotechnology Co., Dalian, China) and tested using nested PCR, targeting the *groEL* gene of *R. prowazekii* and *R. typhi.* Further genetic sequencing confirmed *R. typhi* infection; the GenBank accession number was NC_006142. Based on the test results, a final diagnosis of murine typhus was established. After a 3-week hospitalization, the patient’s condition improved markedly; specifically, the body temperature returned to normal and the patient regained consciousness. A subsequent brain MRI and CT showed a reduction in the number of intracranial lesions and resorption of the cerebral hemorrhage (Fig. 2). The patient was transferred to a local inpatient rehabilitation facility [Brief timeline of events and symptoms have been shown in Table 2].

**Table 1:** Results of laboratory tests

å	Reference Range for Adults	Admission (April 24)	The second day (April 25)	Discharge (May 16)
Hemoglobin (g/L)	131–172	153	150	145
White cell count (per mm^3^)	4.0–10	9.5	12.0	6.2
Differential count (%)				
Neutrophils	50.0–70.0	65.4	61.1	60.9
Lymphocytes	20.0–40.0	29.0	33.3	31.9
Monocytes	3.0–10.0	4.6	4.9	3.6
Eosinophils	0.5–5.0	0.1	0.1	3.4
Basophils	0.0–1.0	0.9	0.6	0.2
C-reactive protein(mg/L)	0.0–8.0	31.8	29.0	5.2
CSF examination				
Cell count				
Red cell count (per mm^3^)			3000	
Nucleated cell count (per mm^3^)			20	
Glucose (mmol/L)	2.5–4.5		2.9	
Chloride (mmol/L)	120–131		134	
Protein(g/L)	0.15–0.45		0.57	
Liver function examination				
Glutamic pyruvic transaminase (U/L)	5–40	58	127	65
Glutamic oxalo-acetic transaminase (U/L)	8–40	82	134	66
Phosphocreatine kinase (U/L)	38–174	257	210	88
Lactate dehydrogenase (U/L)	109–245	522	412	300
Albumin(g/L)				
HBsAg	Negative	Positive		
Syphilis	Negative	Negative		
Anti-HCV	Negative	Negative		
Anti-HIV	Negative	Negative		
TSPOT	Negative	Negative		
Cytomegalovirus antibody(S/CO)				
IgM		-		
IgG		+8.6		
Burkitt’s lymphoma virus antibody (S/CO)				
IgM		+1.1		
IgG		+2.9		
Herpes simplex virus type 1 antibody				
IgG	Negative	Negative		
IgM	Negative	Negative		
Herpes simplex virus type II antibody				
IgG	Negative	Negative		
IgM	Negative	Negative		
Parasitic antibody				
*Paragonimus*	Negative	Negative		
*Schistosoma japonicum*	Negative	Negative		
*Sparganum mansoni*	Negative	Negative		
*Toxoplasma gondii*	Negative	Negative		
*Cysticercus cellulosae*	Negative	Negative		
Hydatid cyst	Negative	Negative		
Serum IgG to rickettsial antibody			(April 28)	(May 12)
*R. typhus*	Negative		1:128	1:2048
*R. conorii*	Negative		Negative	
*O. tsutsugamushi*	Negative		1:32	1:128
*A. phagocytophilum*	Negative		Negative

**Table 2:** Timeline of events and symptoms

Time	Clinical events and symptoms
2014-4-10	Onset of disease
2014-4-20	Date of emergency room visit
2014-4-24	Admission day
2014-4-24	Color Doppler cardiac examination
2014-4-25	Date of lumber puncture
2014-4-26	Initiation of minocycline therapy
2014-4-28	Baseline rickettsial antibody testing
2014-4-28	Parasite antibody testing
2014-5-12	Serial rickettsial antibody testing
2014-5-16	Discharge day

## Discussion and conclusions

In China, *R. typhi* is the leading cause for endemic murine typhus, which is primarily transmitted by the rat flea [7]. Human murine typhus infection is characterized by high fevers, a skin rash, and a combination of a variety of symptoms, such as acute hepatitis, pneumonia, and meningitis. The patient described herein presented with fevers, headaches, dizziness, and coma. Because the patient did not have a rash, which is a typical sign of a rickettsial infection, the condition was initially misdiagnosed at the local hospital.

The unique clinical features of this patient included the following: 1) an elderly hypertensive male with a history of cigarette smoking and alcohol consumption; 2) a 2-week incubation period with a lack of any observable skin rashes and a rapidly deteriorating physical condition; and 3) imaging results that showed multiple cerebral infarctions in the white matter together with intra-cerebral and subarachnoid hemorrhage. On admission, we first ruled out infective endocarditis based on the negative echocardiographic findings. Second, considering the fevers, headaches, elevated C-reactive protein level, and multiple intracranial lesions, meningoencephalitis was thought to be a likely diagnosis. The presence of an eschar together with an insect bite history and positive serum rickettsia-specific antibody tests, led to the final diagnosis of murine typhus complicated by pneumonia, cerebral infarction, and intracranial and subarachnoid hemorrhage.

The correct diagnosis was delayed due to the absence of any skin rashes, although the absence of rashes has been described in 10 % of reported cases [8, 9]. The pathologic features of endemic typhus include systemic vasculitis, which can induce damage to multiple organs and the brain. *Rickettsia typhi* proliferates and spreads via the blood stream causing injury to endothelial and vascular smooth muscle cells, thus resulting in vasculitis. The infection can also involve cerebral arteries and lead to brain damage [10, 11]. Vascular injury is the pathophysiologic basis for meningoencephalitis and a skin rash. The probable mechanism leading to infarction may be caused by small artery spasm or occlusion, and hemorrhage caused by increased vascular permeability due to the infection.

The neuroimaging results correlated with the pathologic findings of cerebral vasculitis, infarctions, and arteriolar thrombonecrosis [12, 13]. Cranial CT scans of rickettsia-infected patients can be normal [14] or show cerebral infarction. MR imaging findings in typhus patients have been previously shown to include focal arterial infarctions, diffuse edema, meningeal enhancement, and the presence of prominent perivascular spaces [12, 15]. In our case, the cranial CT results at the local hospital showed no cerebral infarction or hemorrhage, and the follow-up MR imaging showed multiple white matter lesions, suggesting the presence of infarction, and intracranial and subarachnoid hemorrhage. Similar MR imaging results have been described in patients with cryptococcosis and Lyme disease, which are also characterized by cerebral vasculitis [16, 17]. A study showed that 67 % of patients with abnormal neuroimaging ultimately returned to normal neurologic status and 93 % showed normal imaging after antibiotic treatment [18]. The patient regained consciousness after 2 weeks of antibiotic treatment and was able to walk unassisted after 1 month of therapy.

From the current case and literature review we concludes the following: 1) The medical history and disease evolution are key to the clinical diagnosis of murine typhus. This elderly male farmer presented with a rash that was initially overlooked. 2) *Rickettsia typhi* involving the endothelial and vascular smooth muscle cells resulting in vasculitis not only lead to brain injury, but also cause multisystem organ failure. The imaging studies showed multiple infarctions, edema, and hemorrhage, which are rare in this type of case.3) The proper therapeutic treatment for rickettsial infections is simple and effective with a good prognosis due to early diagnosis.

### Consent

Written informed consent was obtained from the patient for publication of this case report and any accompanying images. A copy of the written consent is available for review by the Editor of this journal.
